# Francesco Amaldi (1939-2024) – a pioneer in RNA research

**DOI:** 10.1038/s44318-025-00388-2

**Published:** 2025-02-27

**Authors:** Irene Bozzoni

**Affiliations:** Sapienza University of Rome and Italian Institute of Technology, Department of Biology and Biotechnologies and Center for Life Nano- & Neuro-Science of IIT, Rome, Italy

Francesco Amaldi died at the age of 84 in Rome on August 13, 2024, assisted by his beloved wife Paola and their children Andrea and Ilaria together with the grandchildren Jaco, Emilio, and Elena. He now rests in peace close to his beloved country house in Rignano Flaminio, near Rome, where he loved to spend his free time dedicating himself to one of his greatest passions: music. He learned to play piano as a child, and later in his life he played also the recorder and ‘viola da gamba’. This, combined with his great manual ability, allowed him over the years not only to enjoy musical moments shared with friends-colleagues but also to handcraft his own viola da gambas. You could feel the same passion he put into discussing the most complex experiments when he described how the different woods should be worked and its surfaces treated for the construction of that instrument. 
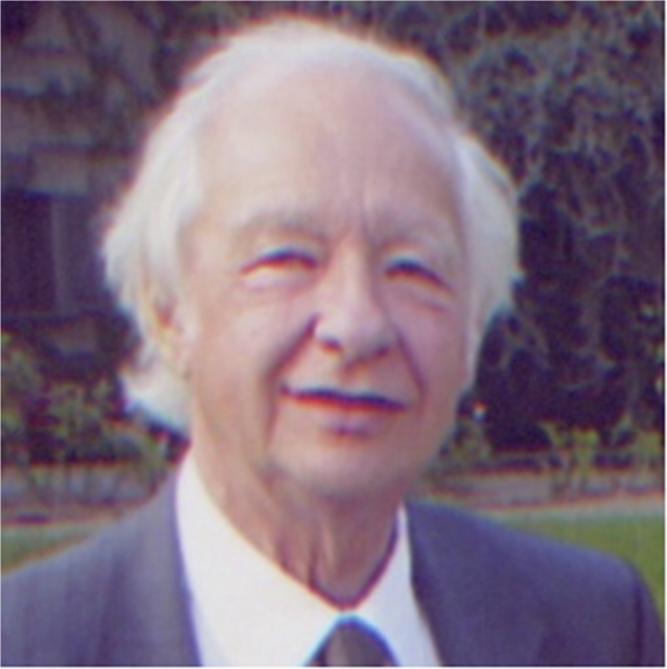


The last few years have been hard for him, a person who has always used his sharp intelligence to approach life in an innovative and creative way, forced instead by a complicated disease that, little by little, resulted in difficulties with his memory and ability to communicate.

His scientific history began in the 60s when he approached molecular biology with the study of tRNA in the bacterium *Streptomyces fradiae* (Trentalance and Amaldi, 1965) but it was consolidated during his post-doc in Giuseppe Attardi's laboratory in Pasadena. A context of great scientific vivacity where people like Max Delbrück, Salvador Luria, Edward Lewis, Seymur Benzer, Eric Davidson, and Roger Sperry laid the foundations of modern biology with a reading based on development and evolution.

His major achievement at that time was the characterization of rRNA base composition and the definition of sequence conservation of rRNA molecules (Amaldi et al, 1969; Amaldi and Attardi, 1971). In parallel, there has been the first identification of the base modification, pseudouridine, in RNA (Amaldi and Attardi, 1968). All that was very pioneering, considering that we were in a pre-sequencing era. However, his most important contribution in the late 60s was the first Cytological hybridization (in parallel with the work of J Gall—Pardue University—and M Birnstiel—Zurich University), that we would now call in situ hybridization, where, together with Mario Buongiorno-Nardelli in Rome, showed the use of ^3^H-labelled rRNA to identify in situ the localization of rDNA genes (Buongiorno-Nardelli M and Amaldi F, 1970).

It was in the following decade that Francesco was quick to spot the newly emerging field of gene cloning and analysis of the fine structure of the genes by making a strong effort to challenge the isolation and cloning of lower abundant transcripts. Also in this case, his enthusiasm for innovation paralleled his ability to gather collaborators to work with him. That is when he managed among the first to produce cDNA and genomic clones for house-keeping genes, successfully accomplished through the use of “hybrid released translation”, a tantalizing methodology. This consisted in the hybridization on single cDNA clones spotted on Millipore filters with pA^+^ mRNA; individual mRNAs were then recovered from the filter and translated in vitro in the presence of S^35^-Met. Correspondence with the respective ribosomal proteins occurred by co-migration on two-dimensional polyacrylamide gels. A rather challenging technique about which Max Birnstiel commented: "... *if you can do it you are very good* ...". Nevertheless, he succeeded and allowed the study of these genes both from a structural and regulatory point of view; in fact, this allowed to discover the presence of intronic regions coding for snoRNA (Loreni et al, 1985) and the regulation of the expression of these genes both at the splicing and translation levels (Bozzoni et al, 1984; Pierandrei-Amaldi et al, 1982).

Amaldi's group was in fact among the first to discover that mRNAs for ribosomal proteins contain a characteristic 5'UTR defined 5'TOP and that this sequence, in combination with the La protein, are involved in translation control in the early stages of Xenopus embryonic development. Moreover, using anucleolate frog mutants, he was also able to demonstrate the tight coupling between the presence of rRNA and the translation of mRNAs for r-proteins (Pierandrei-Amaldi et al, 1985).

In the last years he also extended his analysis to the translational control exerted at the synaptic level being able to set up efficient methods to detect local translation and its regulation (Bagni et al, J Neuroscience, 2000).

After 20 years of work at Sapienza University of Rome, Francesco participated, with a small number of colleagues, in the founding of the second University of Rome “Tor Vergata” where he directed the Department of Biology for many years. Among the countless roles and positions in scientific committees and societies, we recall that he was among the first EMBO members and that he contributed to making EMBO known within the Italian scientific community.

The work led by Francesco Amaldi was not only competitive at the international level but was also relevant to the development of many modern molecular biology techniques. He always had a strong passion for bench work; he was often seen arriving early in the morning in a hurry to start experiments that would be concluded late at night. All methodologies developed in his laboratory had been previously developed by himself, which made them extremely robust and reproducible. His strong international projection opened the doors to important collaborations all over the world, such as those with Robert Perry and Michael Rosbash, and made it possible for his group to have visibility beyond national borders.

While his career was extremely brilliant his personality was very reserved, but this made him even more of a point of reference and gave him an even stronger leadership.

In addition to being an excellent scientist, Francesco Amaldi was an outstanding teacher, a mentor for many students and young researchers who found in him an example of a rigorous and passionate scientist as well as a person of profound human sensitivity and moral integrity. The young collaborators loved him very much because not only did he know how to teach and transmit deep enthusiasm for research but above all for his intellectual honesty in recognizing the credits of each one. His recent textbook on Molecular Biology represents an important legacy for the new generation of biology students. Lastly, Francesco Amaldi was a staunch defender of the autonomy of science and the independence of scientific thought from any political or academic conditioning.
